# Feeling Vital or Fatigued? Relations with Demands and Resources in a University Context

**DOI:** 10.3390/ijerph16162893

**Published:** 2019-08-13

**Authors:** Jan de Jonge, Maria C.W. Peeters, Toon W. Taris

**Affiliations:** 1 Human Performance Management Group, Eindhoven University of Technology, P.O. Box 513, MB, NL-5600 Eindhoven, The Netherlands; 2 Department of Social, Health and Organisational Psychology, Utrecht University, P.O. Box 80140, TC, NL-3508 Utrecht, The Netherlands; 3 School of Psychology, Asia Pacific Centre for Work Health and Safety, University of South Australia, P.O. Box 2471, Adelaide 5001, South Australia

**Keywords:** vitality, vigor, fatigue, demands, resources, DISC Model, job redesign, university staff, university students

## Abstract

This study examines whether specific (matching) combinations of demands and resources exist in the prediction of both positive and negative outcomes (i.e., vitality and fatigue) in a university context. In addition, we test the Demand-Induced Strain Compensation (DISC) Model’s key principles in this context to study its relevance, validity, and generalizability. A cross-sectional survey study was conducted among 397 employees and 497 students at a Dutch university. Hierarchical multiple regression analyses among both employees and students showed matching combinations of demands and resources in the prediction of vitality and fatigue. Specifically, an increase in cognitive demands was particularly associated with more student cognitive vitality when cognitive resources were high. Furthermore, results showed that an increase in cognitive demands was related to *less* cognitive fatigue in both employees and students when cognitive resources were high. Findings partly confirm our hypotheses in showing the important role of matching resources in the relation between demands and vitality and fatigue in university staff and students. Our study reveals that a sustainable work environment is about maintaining a healthy balance between sufficient, matching resources and demands at work or study.

## 1. Introduction

In recent decades, there has been a growing interest in building sustainable organizations [1]. Employers are searching for ways to create a healthy, vital, and sustainable working environment for their employees. We may expect that organizations and management who take care of so-called *human sustainability* will enjoy benefits in attracting and retaining vital employees for lifetime employability and sustainable performance at work [2,3].

Presently, there are still many organizations in which employees work hard in so-called *intensive work systems* with high job demands to maintain productivity while at the same time depleting human, job, and social resources. These systems can have damaging effects in the long run on employees (e.g., in terms of poor health and vitality), as well as on the quality of their products and services [1,4]. In general, it seems that universities can be characterized as intensive work systems for both staff and students, too. Mounting evidence suggests that the level of work stress in universities is high and that it has increased significantly in recent decades (e.g., [5,6,7,8,9,10,11]). This is reflected in the persistent and growing demands of academic life as well as the large number of competing tasks, such as teaching, research, management, seeking funding, valorization efforts, congress visits, and meeting tutorial commitments. Research has identified several key factors that are associated with work stress in university staff. These include work overload, time pressure, lack of job control, lack of social support, lack of promotion prospects and job security, poor levels of reward and recognition, fluctuating roles, increasing levels of red tape and bureaucracy, poor leadership and management, lack of funding, financial resources and support services, and intense student interactions [5,7,9,12]. Not surprisingly, high work stress in university staff caused by these work stressors can lead to poor health (e.g., mental and physical fatigue), reduced vitality, sickness absence, and poor performance [5,9,10].

Work stress in universities does not only impact their employees. It also has consequences for their students, too. Although, strictly speaking, students are not employed, their core activities can also be considered to be “work” [13,14,15]. Like many paid workers, students work in hierarchical structures with specific “job” demands and variable levels of “job” resources such as control and social support (e.g., [13,14,16,17]). They are required to attend classes, to complete assignments and exams, to meet deadlines, and study progress is dependent on performance [15]. Research has consistently shown increasing levels of stress in university students (e.g., [11,13,14,18,19]). For instance, a survey among 1093 UK university students found that 87% of them reported stress at their work, and the factors that triggered their work stress most were course workload, deadlines, exams, study–home balance, and grades/performance pressure [18]. A relatively large survey study among 4077 Dutch university students showed that 56% of them reported high or extremely high work pressure [19]. Furthermore, there is a growing trend for university students to combine their studies with paid employment in an effort to bridge the gap between study costs and financial resources. Obviously, this has increased students’ total working hours [20] and work demands [16] to some extent, too. However, stress among students has not often been approached using a theoretical framework. To our knowledge, only two empirical studies used such a framework in university students. Applying Karasek and Theorell’s Demand–Control–Support Model [21], these studies showed that—similar to other work contexts—psychological distress was predicted by high demands, low control, and low social support [13,14]. Therefore, in line with this research, we consider what students do at a university as being similar to the tasks that make up an academic job. This implies that we can examine theorized links between both employees’ and students’ work environment, health, well-being, and performance.

Several theoretical frameworks have been advanced to explain the role of job resources in the relation between job demands and job-related outcomes such as health and well-being [22]. Most of these frameworks propose additive and moderating effects of job demands and job resources in the prediction of health and well-being outcomes. Specifically, additive effect models assume job demands and job resources to impact independently on job-related outcomes, whereas moderating effect models propose that resources moderate the relation between demands and outcomes. While there is ample empirical evidence for additive effect models, moderating effect models have received mixed support at best [22,23]. There is at least one important reason so many researchers have failed to find moderating effects. Early research has treated job demands and job resources as global and unidimensional constructs, considering the job characteristics within each category as being largely interchangeable, thus obscuring the differential impact of specific components [24]. In reaction to this view, several researchers have argued that the associations among demands, resources, and outcomes depend on the respective type of demands, resources, and outcomes (e.g., [25,26]). In particular, researchers have proposed that specific kinds of demands, resources, and outcomes should match to show moderating effects in the prediction of job-related outcomes. This theoretical refinement has been incorporated into the so-called Demand-Induced Strain Compensation (DISC) Model [27,28,29], which will be discussed in more detail below.

To conclude, both university staff and university students suffer from increased stress at work. They must deal with increasing demands (e.g., quantitative and qualitative workload) to maintain their performance norms, while simultaneously having a high chance of depleting their resources. The aim of the present study is, accordingly, two-fold. First, this study examines specific (matching) combinations of demands and resources in the prediction of both positive and negative job-related outcomes (i.e., vitality and fatigue) in a sample of university employees and students. Second, we test the DISC Model’s key principles in a university context to study its relevance, validity, and generalizability.

### The Demand-Induced Strain Compensation Model

**Figure 1:**
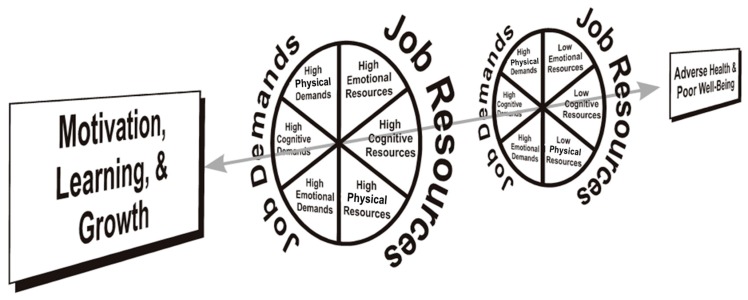
Demand-Induced Strain Compensation (DISC) Model.

The DISC Model, as depicted in Figure 1, claims that stress-buffering and motivating effects of job resources largely depend on the so-called “match” or “fit” between specific types of job demands and job resources [27,28,29]. Job demands refer to those properties of the job that require immediate or sustained cognitive, emotional and/or physical effort. Job resources are conceptualized as work-related assets that can be employed when somebody must deal with demands at work.

The DISC Model entails two main principles: the multidimensionality principle and the triple-match principle. The *multidimensionality principle* claims that job demands, job resources, and work-related outcomes are multidimensional constructs that contain cognitive, emotional, and physical components. As far as job demands are concerned, three types can be distinguished: (1) cognitive demands that impinge primarily on human information processing; (2) emotional demands, mainly concerning the effort needed to deal with desired emotions during interpersonal transactions; and (3) physical demands that are primarily associated with the musculoskeletal system. Similarly, job resources may have a cognitive–informational component (e.g., job control, colleagues or computer systems providing information), an emotional component (e.g., colleagues providing sympathy, affection, and a listening ear) and a physical component (e.g., instrumental help from colleagues or ergonomic aids). Finally, similar to demands and resources, job-related health, well-being, and performance-based outcomes may also comprise cognitive, emotional, and physical dimensions. These outcomes can be either negative or positive. For instance, concentration problems and creativity represent cognitively laden outcomes, emotional exhaustion and emotional energy represent emotionally laden outcomes, and physical health complaints and physical strength mainly reflect physical outcomes.

The second main principle is the *triple-match principle* (TMP). The TMP proposes that the strongest, interactive relations between job demands and job resources are observed if demands and resources and outcomes are based on qualitatively identical dimensions. For instance, emotional support from colleagues is most likely to moderate (i.e., mitigate) the relation between emotional demands (e.g., irate clients) and emotional outcomes (e.g., emotional exhaustion). Conversely, it is more difficult to see why high emotional support should moderate the relation between physical demands and cognitive outcomes. Therefore, the TMP suggests not only that job demands and job resources should match, but also that both job demands and job resources should match job-related outcomes. As a theoretical basis for the matching hypothesis, functional homeostatic regulation is proposed, which can easily be applied to organizational settings [30]. In short, functional homeostatic regulation at work involves self-regulation processes to cope with states of psychological imbalance induced by job demands. Ideally, people will activate functional, matching job resources to mitigate the effects of specific job demands.

In line with recent developments in occupational health psychology, the model further predicts that high job demands stimulate positive psychological or physiological states best as long as employees possess sufficient functional, corresponding kinds of job resources (e.g., [27,31,32]). In the case employees possess high levels of resources, they can try out different ways of dealing with job demands. Consequently, learning and growth will result [31]. For instance, cognitively challenging work tasks (e.g., making difficult decisions) in combination with sufficient cognitive job resources (e.g., job control) may stimulate active learning, vitality, and creativity.

Generally, it is proposed that workers will first deal with their job demands using easily available matching job resources. However, if such matching job resources are not available, or when they are depleted [33], employees will search for other, less-matching job resources and will even use job resources that do not correspond to their job demands [34]. Thus, next to triple-matches, the DISC Model also distinguishes “lower order” matches which in general are weaker and thus less likely to occur [28]. For instance, although it is assumed that emotional demands are most likely to affect emotional outcomes, there may also be an association between emotional demands and cognitive outcomes that could be moderated by emotional resources [27]. This idea of match is referred to as a *double-match of common kind* because there is a match between job demands and job resources, while job-related outcomes manifest at a different dimension. Similarly, there could also be a double-match between demands and outcomes when resources are a sign of a different dimension (e.g., an association between emotional demands and emotional outcomes that is moderated by cognitive resources), or a double-match between resources and outcomes when demands manifest at a different dimension (e.g., an association between emotional demands and physical outcomes that is moderated by physical resources). This idea of match is referred to as a *double-match of extended kind* as it goes beyond what is commonly proposed in the literature [35]. Finally, moderating effects are least likely to occur (or do not occur at all) in cases of non-match—i.e., when demands, resources, and outcomes all constitute a different component (e.g., an association between emotional demands and cognitive outcomes that is moderated by physical resources).

In this study, we tested the model’s TMP using both a positive and a negative job-related outcome: vitality and fatigue. We chose these outcomes as they can be considered key markers of building sustainable organizations [1,36]. Vitality was represented by Shirom’s [37] concept of vigor, consisting of cognitive liveliness, emotional energy, and physical strength. Fatigue was represented by Stein and colleagues’ [38] multidimensional fatigue concept, consisting of cognitive, emotional, and physical fatigue. The two key principles of the DISC Model are guided by two further corollaries pertaining to balance (left wing of Figure 1) and compensation mechanisms (right wing of Figure 1). First, the *balance* or *activation-enhancing mechanism* proposes optimal conditions for employee motivation, learning, creativity, vitality, and growth if there is a balanced mixture of high job demands and high matching job resources. For instance, employees who need to solve complex problems are most likely to become creative if they have sufficient cognitive resources (e.g., instant access to information or authority to decide the work method themselves) to deal with their cognitively demanding tasks. Consequently, we expect that:

**Hypothesis** **1:** *Higher cognitive demands are associated with higher cognitive liveliness, and this relation is moderated (i.e., strengthened) by matching cognitive resources.*

**Hypothesis** **2:** *Higher emotional demands are associated with higher emotional energy, and this relation is moderated (i.e., strengthened) by matching emotional resources.*

**Hypothesis** **3:** *Higher physical demands are associated with higher physical strength, and this relation is moderated (i.e., strengthened) by matching physical resources.*

Second, the *compensation* or *stress-buffering mechanism* proposes that the adverse effects of high job demands on employee health and well-being can be counteracted if employees have sufficient job resources to deal with their demanding work tasks. As explained before, job resources from the same conceptual domain as job demands are most likely to counteract these negative effects. Consequently, we formulated the following hypotheses:

**Hypothesis** **4:** *Higher cognitive demands are associated with higher cognitive fatigue, and this relation is moderated (i.e., buffered) by matching cognitive resources.*

**Hypothesis** **5:** *Higher emotional demands are associated with higher emotional fatigue, and this relation is moderated (i.e., buffered) by matching emotional resources.*

**Hypothesis** **6:** *Higher physical demands are associated with higher physical fatigue, and this relation is moderated (i.e., buffered) by matching physical resources.*

The DISC Model has been tested in various research samples in many different countries [22]. Different kinds of employees were subjects of study, though most of them are human-services workers such as nursing, retail, and teaching staff. Van den Tooren and colleagues [39] conducted a review study of 29 DISC studies to investigate the empirical evidence for the key assumption of the model; that is, the triple-match principle. Results showed that the TMP was largely supported. Specifically, there were 32 significant demand–resource interactions out of 108 tested triple-match interactions (29.6%), 36 out of 327 tested double-matching interactions (11.0%), and 6 out of 76 tested non-matching interactions (7.9%). Please note that the latter percentage is close to what can be expected by chance combined with publication bias (i.e., the tendency to publish studies supporting the hypotheses more often than studies showing null or negative findings). This review suggested that matching job resources are more functional than less-matching and non-matching job resources to deal with specific types of job demands. In line with these findings, our final hypothesis is:

**Hypothesis** **7:** Triple-match interactions are most likely to be found, followed by double-match interactions (both common kind and extended kind), and non-matching interactions are least likely to occur.

## 2. Materials and Methods

### 2.1. Study Design, Data, and Procedure

We conducted a cross-sectional survey study in which participants had to fill out an online questionnaire, which can be obtained from the corresponding author upon request. All employees and students of a Dutch university (approximately 14,500 people) received an email with a brief description of the survey, its objectives, and a corresponding weblink. The online survey questionnaire was developed in Unipark Software. Participants could fill out the survey by using either a desktop, laptop, tablet, or smartphone. 

In total, 1831 people accessed the online survey by clicking the weblink they received. After removing the participants that did not finish the survey, the total sample included 894 people with a survey completion rate of 48.8%. Concerning the participants’ status, 55.6% of the participants were students (497 people), whereas 44.4% of the participants (397 people) were university employees (i.e., 254 people as academic staff and 143 people as non-academic staff). More than half of the employees (51.6%) were male, whereas 62.4% of the students were male (513 men and 377 women in total; four people did not fill out their gender). Mean age of employees was 35.2 years (SD = 13.9), and mean age of students was 21.3 years (SD = 6.2). As far as education is concerned, 31.0% of the participants held a high school degree (277 people), 24.4% held a BSc degree (218 people), 26.2% held a MSc degree (234 people), and 10.7% held a PhD degree (96 people). Moreover, 63 participants (7%) held an undergraduate or a college degree, and 2 persons (0.2%) had an elementary school level of education. All participants gave their informed consent for inclusion before they participated in the study. The study received institutional approval and was conducted in accordance with ethical principles of the Declaration of Helsinki and the American Psychological Association.

### 2.2. Variables and Instruments

#### 2.2.1. Demands and Resources at Work/Study

Cognitive, emotional, and physical demands and resources were measured with the DISC Questionnaire 2.1 (DISQ 2.1; [40]). Different versions of this international and widely used questionnaire have demonstrated good psychometric properties in different occupational groups (e.g., [41]). Each DISQ scale consists of five items, except for the emotional demands and cognitive resources scales, which both have six items. All items were rated on a 5-point frequency scale ranging from 1 (never or very rarely) to 5 (very often or always). Examples of the items for demands are “I need to display high levels of concentration and precision at work/study” (cognitive; Cronbach’s α = 0.79), “I have to do a lot of emotionally draining work” (emotional; Cronbach’s α = 0.83), and “I have to perform a lot of physically strenuous tasks to carry out my (study) job” (physical; Cronbach’s α = 0.84). Example items of resources are “I have the opportunity to determine my own work method” (cognitive; Cronbach’s α = 0.81), “I receive emotional support from others (e.g., clients, colleagues, or supervisors) when a threatening situation at work/study occurs” (emotional; Cronbach’s α = 0.83), and “I am able to use adequate technical equipment to accomplish physically strenuous tasks” (physical; Cronbach’s α = 0.88).

#### 2.2.2. Employee/Student Vitality and Fatigue

We used the Shirom–Melamed Vigor Measure (SMVM) that was empirically validated in more than 20 empirical studies in different countries [37]. The SMVM measures the three different components of vigor among participants; that is, three items for cognitive liveliness, four items for emotional energy and five items for physical strength. Specifically, participants had to rate how often they displayed each feeling during the period of one month on a 7-point Likert scale, ranging from 1 (almost never) to 7 (almost always). An example item of cognitive liveliness is “I feel I can think rapidly” (Cronbach’s α = 0.85). Regarding emotional energy, an example item is “I feel capable of being sympathetic to people (e.g., students, colleagues, customers)” (Cronbach’s α = 0.91). Finally, an example item for physical strength is “I feel full of energy” (Cronbach’s α = 0.93).

In addition, we used the Multidimensional Fatigue Symptom Inventory—Short Form (MFSI—SF) developed by Stein and colleagues [38]. This measure reflects three different types of fatigue: cognitive, emotional, and physical fatigue. Participants had to evaluate their agreement with 18 statements (i.e., six items per construct) during the period of one month, on a 5-point Likert scale ranging from 1 (not at all) to 5 (extremely). An example item for cognitive fatigue is: “I have trouble remembering things” (Cronbach’s α = 0.86). With respect to emotional fatigue, an example item is “I feel upset” (Cronbach’s α = 0.91). Finally, an example of physical fatigue is “My muscles ache” (Cronbach’s α = 0.82).

#### 2.2.3. Demographics

Demographics used in this study are gender (0 = male; 1 = female), age (years) and educational level (1 = low to 7 = high). They were used as control variables as they appeared to be potentially important in former research [6,8,11,42,43] as well as significantly associated with our outcome measures. For instance, both a systematic literature review and a meta-analysis on university students’ stress showed gender differences [8,11], while stress research among university employees revealed gender, age and educational differences (e.g., [6,7,9,10]).

### 2.3. Statistical Analysis

First, Pearson zero-order correlational analyses were conducted to obtain an initial overview of our survey data. Second, hierarchical multiple regression analyses (HMRAs) were used to examine the relation between (1) demands and resources, and (2) the six outcome measures. All analyses were performed in IBM SPSS Statistics 25 (SPSS Inc., Chicago, IL, USA).

The HMRAs were conducted with simultaneous entry of variables within each hierarchical step. Accordingly, we performed several hierarchical modeling steps. In the first step, the demographic variables and the standardized main terms of demands and resources were included. Postulated moderating effects were tested by adding multiplicative interaction terms (demands × resources) of standardized demands and resources into the second step of the HMRAs [44]. However, due to the large number of possible interaction effects in one single analysis we decided to split the analysis. According to the theoretical assumptions of the DISC Model, we split the analyses into matching and non-matching demand–resource interaction testing [28,45]. Therefore, in the first analysis, we simultaneously tested 3 triple-matches and 6 double-matches of common kind for successively vitality and fatigue. The second analysis included the remaining match and non-match conditions. In this case, we simultaneously tested 12 double-matches of extended kind and 6 non-matches for each outcome measure, respectively. If a statistically significant moderating effect is in line with the DISC Model’s principles, it is called a theoretically valid triple-match, double-match, or non-match. If a statistically significant moderating effect contradicts the model’s principles, it is called a theoretically nonvalid or reversed triple-match, double-match, or non-match [39]. Furthermore, significant moderating effects were graphically represented [44], and a test of slope significance of the respective simple regression lines was carried out [46]. Please note that only graphical representations of significant triple-match interactions were shown due to space restrictions. 

The final step was to decide if it would be necessary to split the sample in two subgroups, (i.e., one for employees and one for students), and to conduct subgroup analyses accordingly. To do so, a dummy variable depicting employees’ or students’ subgroup was created (0 = employees and 1 = students) and entered in each regression model, along with the whole set of control and standardized independent variables. Twelve tests were performed in total, one for each outcome measure and each regression model. Five out of these 12 tests (42%; four for vitality and one for fatigue) showed significantly different regression models between the two subgroups. Therefore, subgroup analysis for both employees and students was empirically justified to be carried out.

## 3. Results

A first inspection of the Pearson zero-order correlations in Table 1 shows that all demands were positively associated with the three fatigue outcomes. Next, cognitive demands were positively related to all vitality outcomes. Finally, all resources were positively associated with all vitality outcomes, whereas cognitive and emotional resources were negatively related to all fatigue outcomes. 

### 3.1. Demands and Resources as Predictors of Vitality

In line with our first three hypotheses, we tested all demands and resources (interactions inclusive) as predictors of both employee and student vitality (see Table 2 and Table 3).

#### 3.1.1. Predictors of Employee Vitality

Table 2 shows the HMRA results for the tests of triple-matches and double-matches of common kind interactions between demands and resources in the employee subgroup. Regarding employee vitality, one interaction model and two main effect models were significant. Please note that no interaction effects are displayed as far as main effect models were concerned. The interaction model concerns emotional energy. Specifically, one double-match of common kind occurred, involving the interaction of physical demands and physical resources in prediction of emotional energy (*b* = −0.15, *p* = 0.04). Simple slope tests showed a significant slope for high physical resources. This interaction effect shows that an increase in physical demands was related to less emotional energy in the case of high physical resources (+1 SD; *t* = −2.94, *p* = 0.004). Furthermore, there was no association between physical demands and emotional energy in the case of low physical resources (−1 SD; *t* = 0.92, *p* = 0.356). In general, the shape of this double-match of common kind interaction was against the DISC Model’s assumptions. Therefore, it is characterized as a reversed or nonvalid interaction effect.

The remaining outcomes of employee vitality showed only two main effect models to be significant. Remarkably, identical predictors were significant for both cognitive liveliness and physical strength. Specifically, cognitive demands, cognitive resources, and emotional resources were positively related to both vitality outcomes, whereas emotional demands and physical resources were negatively associated with cognitive liveliness and physical strength, respectively. Overall, the explained variance (*R*^2^) was 0.28 for cognitive liveliness, 0.25 for emotional energy, and 0.24 for physical strength.

#### 3.1.2. Predictors of Student Vitality

Table 3 shows the HMRA results from testing triple-matches and double-matches of common kind interactions between demands and resources in the student subgroup. Regarding student vitality, one out of three interaction models was significant. Specifically, the interaction model concerns the triple-match of cognitive demands and cognitive resources (*b* = 0.08, *p* = 0.02) as well as a double-match of common kind of physical demands and physical resources (*b* = 0.18, *p* = 0.01) in the prediction of cognitive liveliness.

**Figure 2:**
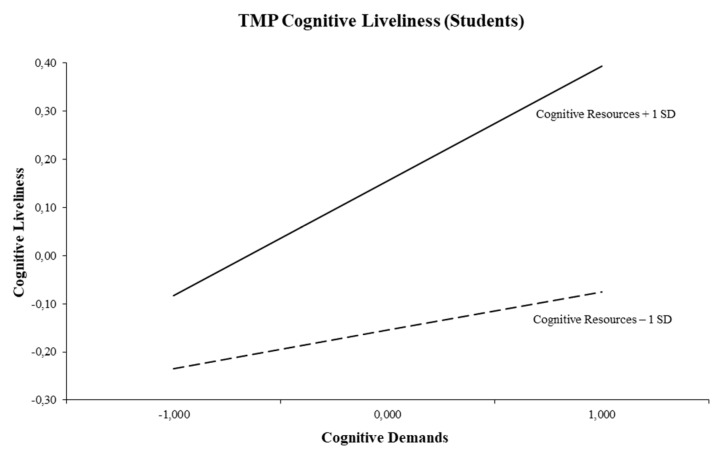
Interaction between cognitive demands and cognitive resources for cognitive liveliness (Students).

Simple slope tests for the triple-match interaction revealed a significant slope for high cognitive resources. Figure 2 shows that an increase in cognitive demands was particularly associated with more cognitive liveliness in the case of high cognitive resources (+1 SD; *t* = 3.07, *p* = 0.002). This is called a synergistic interaction effect [47]. In the case of low cognitive resources (−1 SD; *t* = 1.03, *p* = 0.302), cognitive demands were not related to cognitive liveliness. Furthermore, simple slope tests for the double-match interaction showed a significant slope for high physical resources. Specifically, this interaction effect shows that an increase in physical demands was related to more cognitive liveliness when physical resources were high (+1 SD; *t* = 3.19, *p* = 0.002). Physical demands were not associated with cognitive liveliness in case physical resources were low (−1 SD; *t* = −1.36, *p* = 0.176). Regarding the second dimension of student vitality, emotional energy, only the main effect model appeared to be significant. This model shows that both cognitive demands and emotional resources were positively related to emotional energy. The main effect model of the third dimension of student vitality, physical strength, indicates five predictor variables to be significant. More specifically, cognitive and physical demands were positively related to physical strength. Whereas emotional demands were negatively related to physical strength. Furthermore, both cognitive and emotional resources were positively associated with physical strength. Overall, *R*^2^s were 0.15 for cognitive liveliness, 0.18 for emotional energy, and 0.17 for physical strength.

### 3.2. Demands and Resources as Predictors of Fatigue

In line with Hypotheses 4 to 6, we tested all demands and resources (interactions inclusive) as predictors of both employee and student fatigue (see Table 2 and Table 3).

#### 3.2.1. Predictors of Employee Fatigue

**Figure 3:**
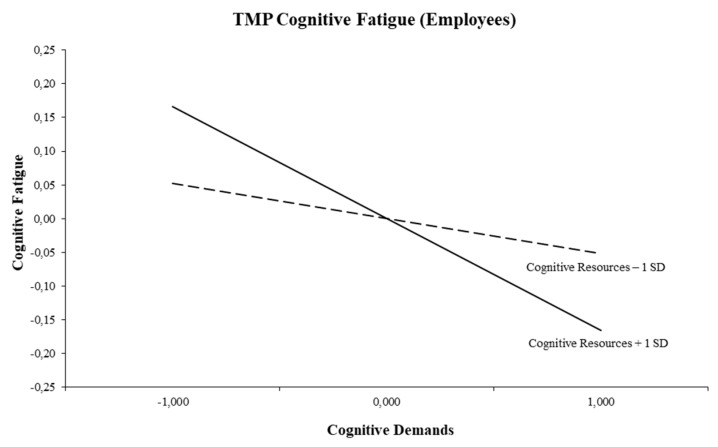
Interaction between cognitive demands and cognitive resources for cognitive fatigue (Employees).

With respect to employee fatigue, Table 2 shows that all three interaction models are significant. First, the triple-match interaction of cognitive demands and cognitive resources in the prediction of cognitive fatigue was significant (*b* = −0.06, *p* = 0.01). The graphical representation can be found in Figure 3, in which the simple slope for high cognitive resources was significantly different from zero. The figure shows that an increase in cognitive demands was associated with less cognitive fatigue in the case of high cognitive resources (+1 SD; *t* = −2.67, *p* = 0.008). There was no relation between cognitive demands and cognitive fatigue when cognitive resources were low (−1 SD; *t* = −0.81, *p* = 0.420). 

Furthermore, a double-match of common kind interaction was significant; that is, the interaction of physical demands with physical resources to predict cognitive fatigue (*b* = 0.12, *p* = 0.03). Simple slope tests showed a significant slope for high cognitive resources. This interaction effect shows that high physical demands were related to high cognitive fatigue when physical resources were at a high level (+1 SD; *t* = 3.13, *p* = 0.002). In the case of low physical resources (−1 SD; *t* = −0.61, *p* = 0.541), physical demands were not related to cognitive fatigue. Again, the shape of this double-match of common kind interaction is against the DISC Model’s assumptions, and is characterized as a nonvalid interaction effect.

Second, two double-matches of common kind interactions occurred regarding emotional fatigue as an outcome. The first interaction was that of cognitive demands and cognitive resources in the prediction of emotional fatigue (*b* = −0.06, *p* = 0.01). Simple slope tests proved that both slopes were not significantly different from zero. Therefore, cognitive demands were not associated with emotional fatigue; neither in the case of high cognitive resources (+1 SD; *t* = −1.41, *p* = 0.163) nor in the case of low cognitive resources (−1 SD; *t* = 0.36, *p* = 0.721). The second interaction was that of physical demands with physical resources to predict emotional fatigue as well (*b* = 0.11, *p* = 0.03). Simple slope tests showed a significant slope for high physical resources. The corresponding interaction effect shows that if physical resources were high (+1 SD; *t* = 2.27, *p* = 0.024), high physical demands were related to high emotional fatigue. Physical demands were not associated with emotional fatigue in the case of low physical resources (−1 SD; *t* = −1.18, *p* = 0.241). As this result ran counter to standard stress theory as assumed by the DISC Model, it is characterized as a reversed, nonvalid interaction effect. 

Third, a double-match of common kind interaction occurred with respect to the third dimension of fatigue; that is, physical fatigue. This interaction effect is that of cognitive demands and cognitive resources in the prediction of physical fatigue (*b* = −0.05, *p* = 0.03). Simple slope tests revealed that both slopes were not significantly differed from zero. To be more specific, cognitive demands were not related to physical fatigue; neither in the case of high cognitive resources (+1 SD; *t* = −1.17, *p* = 0.242) nor in the case of low cognitive resources (−1 SD; *t* = 0.51, *p* = 0.611). Finally, *R*^2^s were 0.20, 0.30 and 0.16 for cognitive, emotional, and physical fatigue, respectively.

#### 3.2.2. Predictors of Student Fatigue

**Figure 4:**
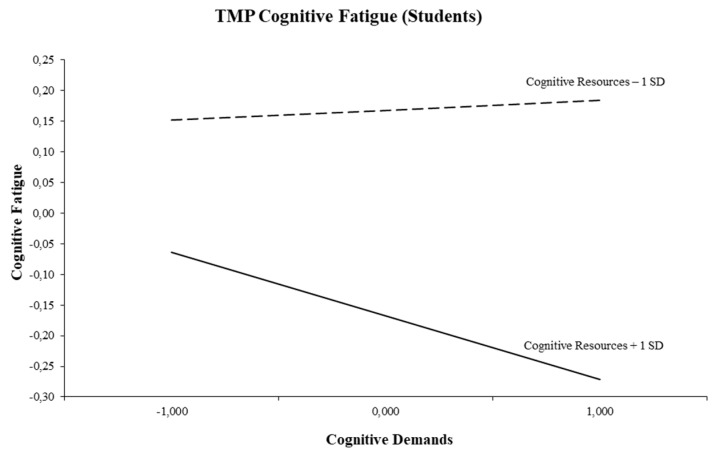
Interaction between cognitive demands and cognitive resources for cognitive fatigue (Students).

We found all three interaction models to be significant for student fatigue (see Table 3). Two interaction models showed the expected triple-match interactions for successively cognitive fatigue (*b* = −0.06, *p* = 0.01) and emotional fatigue (*b* = −0.11, *p* = 0.003). Figure 4 presents the triple-match interaction between cognitive demands and cognitive resources in the prediction of cognitive fatigue. Simple slope tests showed a significant slope for high cognitive resources. The figure indicates that high cognitive demands were associated with less cognitive fatigue when cognitive resources were high (+1 SD; *t* = −2.00, *p* = 0.046). Cognitive demands were not related to cognitive fatigue when cognitive resources were low (−1 SD; *t* = 0.31, *p* = 0.760). 

**Figure 5:**
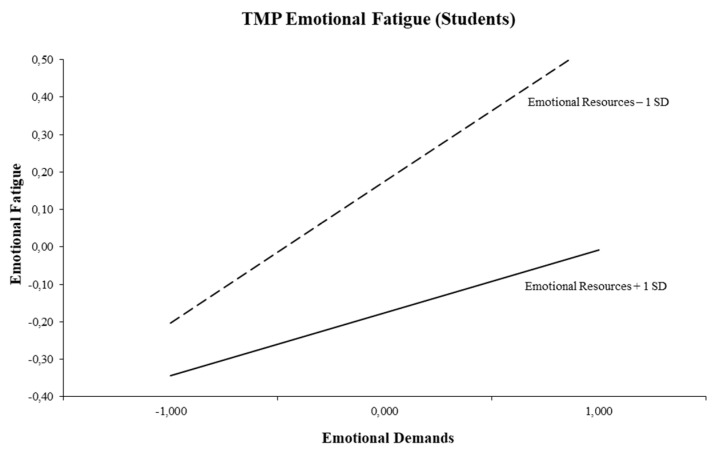
Interaction between emotional demands and emotional resources for emotional fatigue (Students).

The triple-match interaction of emotional demands and emotional resources in the prediction of emotional fatigue is depicted in Figure 5. Simple slope tests proved that both regression slopes are significantly different from zero. As can be seen, an increase in emotional demands was associated with more emotional fatigue both at low levels of emotional resources (−1 SD; *t* = 2.66, *p* = 0.008) and at high levels of emotional resources (+1 SD; *t* = 5.98, *p* = 0.000). However, at high levels of emotional resources, the positive association between emotional demands and emotional fatigue became weakened.

Although the interaction model regarding physical fatigue was significant, the three separate demand–resource interaction effects were not. Overall, it appears that emotional demands were positively related to physical fatigue, whereas cognitive and emotional resources were negatively associated with physical fatigue in the student group. Finally, *R*^2^s were 0.15, 0.25 and 0.13 for cognitive, emotional, and physical fatigue, respectively.

### 3.3. Remaining Demand–Resource Interactions as Predictors of Vitality and Fatigue

The remaining demand–resource interactions (i.e., double-match of extended kind and non-match) were also tested in the prediction of vitality and fatigue in both subgroups. Appendix A depicts the corresponding HMRA findings for employees and students, respectively. For employees, Table A1 generally shows 3 out of 24 significant double-match of extended kind interaction effects (12.5%), along with 1 out of 12 significant non-match interaction effect (9.0%). For students, Table A2 generally shows 4 out of 24 significant double-match of extended kind interactions (17.0%), and 2 out of 12 significant non-match interactions (17.0%). For both groups, explained variance ranged from *R*^2^ = 0.13 (physical fatigue of students) to *R*^2^ = 0.29 (emotional fatigue of employees).

### 3.4. Demand–Resource Interactions and the Degree of Match

Regarding Hypothesis 7, Table 4 shows the combined pattern of demand–resource interactions and the degree of match in both subgroups as well as in the whole sample. In total, we found 4 significant out of 12 triple-match interactions (33.3% valid interactions), 8 (and 5 reversed) out of 72 tested double-match interactions (11.1% valid interactions), and 1 (and 2 reversed) out of 24 tested non-match interactions (4.2% valid interactions). Initially, the valid percentage (i.e., interactions that conformed to the DISC Model’s principles and stress theory in general) of significant interaction effects found appeared to be progressively related to the degree of match in both subgroups as well as in the total sample. 

To statistically test this assumption in the whole sample, we computed a Pearson correlation coefficient between the regression weights of all interaction effects (108 Bs in total, consisting of 12 triple-matches, 72 double-matches and 24 non-matches) and the degree of match (1 = non-match; 2 = double-match; 3 = triple-match) as suggested by Dormann (personal communication, 21 March 2018). A significant correlation between the Bs and the degree of match would support the conclusion that match adds predictive validity. The corresponding analysis showed that there was a significant positive association between the regression weights and the degree of match (*r* = 0.16, *p* = 0.048). In other words, a higher degree of match was related to stronger regression weights of the interactions.

## 4. Discussion

This study examined whether specific (matching) combinations between demands and resources existed in the prediction of vitality and fatigue in a cross-sectional sample of university staff and students. By doing so, we examined the relevance, validity, and generalizability of the DISC Model’s key principles in a university context which, to our knowledge, has never been done before. 

### 4.1. Theoretical Implications

#### 4.1.1. Predictors of Vitality

Regarding vitality, analyses showed that only our first hypothesis was confirmed (student subgroup only) and thus no empirical support for Hypotheses 2 and 3 was found. An increase in cognitive demands was particularly associated with more cognitive liveliness in the case of high cognitive resources, which is in line with the DISC Model’s balance or activation-enhancing mechanism [27,29] and with vitality research (e.g., [1,36,37]). In fact, the balance principle suggests a strengthening rather than a buffering effect of resources. Therefore, students are likely to experience cognitive liveliness if they have sufficient cognitive resources (e.g., control at work or information access) to deal with their cognitively demanding tasks. Though we did find a double-match interaction between physical demands and physical resources, it predicted a different vitality outcome. This double-match interaction in the prediction of cognitive liveliness suggests that—next to a cognitive triple-match interaction—also a double-match interaction arising from another domain (i.e., physical) can be functional for maintaining students’ cognitive liveliness. In other words, students are also likely to experience cognitive liveliness if they have sufficient physical resources (e.g., ergonomic aids) to deal with physically demanding tasks. These findings correspond to the outcomes of a review study by Van den Tooren and colleagues [39], which showed that valid activation-enhancing effects were equally likely to occur in the case of matching cognitive demands/resources and matching physical demands/resources.

We did not find empirical support for valid (i.e., matching) interactions in the prediction of employee vitality (i.e., no support for Hypotheses 1 to 3). Only one double-match interaction between physical demands and physical resources was significant, but predicting a different outcome (i.e., emotional energy). Moreover, this interaction went against the DISC Model’s assumptions, and was characterized as a nonvalid interaction effect. However, the main effect models showed several predictors in line with the matching principle, but they were not able to show significant, synergistic, interaction effects. Therefore, for employees, job demands and job resources impact independently on employee vitality. This agrees with earlier DISC research showing more support for the stress-buffering mechanism than the activation-enhancing mechanism [22,39]. An explanation for this could be that for employees, demands at work are seen more as hindrance demands, whereas for students, they are seen as challenge demands, too [48]. Possibly, job resources are particularly functional as stress buffers for employees, but not as activation-enhancers (as is the case for students).

#### 4.1.2. Predictors of Fatigue

With respect to fatigue, more promising results emerged. Hypothesis 4 was confirmed in both employees and students. Indeed, the two triple-match interactions show that an increase in cognitive demands was related to less cognitive fatigue in both employees and students when cognitive resources were high. This result is in line with the compensation or stress-buffering principle of the DISC Model, which describes the self-regulating processes where cognitive resources such as control or information access are used to diminish the negative effects of cognitive demands on cognitive fatigue. These triple-match findings are also in accordance with a longitudinal study among Belgian employees in the technology sector [49]. The fifth hypothesis was confirmed in the student subgroup only. We found that an increase in emotional demands was related to less emotional fatigue in the case emotional resources were high. This is also congruent with numerous empirical studies in which the buffering role of emotional resources has been shown (e.g., [28,30,39,45,50]). Again, this is in accordance with the DISC Model’s compensation principle [22]. To put it differently, students are likely to experience less emotional fatigue if they have sufficient emotional resources such as emotional support from other students, staff, or family to deal with emotionally demanding tasks and problems.

Hypothesis 6 was not confirmed; neither in the employee subgroup nor in the student subgroup. Apparently, physical resources were not able to play a buffering role in the relation between physical demands and physical fatigue. An explanation for this is that both physical demands and physical fatigue were relatively low in our study sample (see also Table 1) compared to, for instance, health care workers or construction workers [39]. Therefore, in most cases, physical resources were not necessary to play a buffering role for physical fatigue. In the case that they played a role at all, it was only a main effect function in the employee subgroup. In other words, for university staff, an increase in physical resources, such as a helping hand or ergonomic aids, was related to a decrease in physical fatigue.

We found several double-match interactions of common kind in the employee subgroup. Two of them were against the DISC Model’s assumptions, and were characterized as nonvalid interaction effects. Nevertheless, the two valid double-match interactions in the prediction of successively emotional and physical fatigue suggest that a double-match interaction arising from another domain (i.e., cognitive demands and resources) can also be functional for diminishing employees’ emotional and physical fatigue [39]. Overall, a good balance between cognitive demands and cognitive resources seems to be beneficial for all dimensions of employee fatigue. One explanation for the dominant role of cognitive job characteristics in this respect may be that people usually operate (i.e., make decisions, take action) from a set of cognitive schemes or frames of reference [51]. The specific values, beliefs, needs, and understandings that stem from these frames of reference may make people highly sensitive to detect and use particular affordances in the workplace [52,53]. Affordances are properties of the work environment that can exert an influence on the person only if s/he possesses the complementary characteristic to make use of a certain affordance. For instance, health care workers are often involved in emotion work, and may be particularly alert to sources of emotional support. In the current study, respondents’ academic environment and, hence, involvement in cognitive work may have made them alert to sources of knowledge, information, and expertise. In other words, our respondents may have operated from a frame of reference that made them more sensitive to cognitive job demands and resources [54].

Across the board, the explained variance (*R*^2^s) was higher in the employee subgroup compared to the student subgroup for both vitality and fatigue. Even though the DISC Model can be applied to students as well, it seems to be a better explanatory theoretical framework for employees than for students. Notwithstanding the latter, the *R*^2^s found are in line with other DISC research (e.g., [30,39,42]).

#### 4.1.3. Demand–Resource Interactions and the Degree of Match

Finally, we investigated if the likelihood of finding triple-match interactions is greater than the likelihood of finding double-match interactions (of common and of extended kind) and non-matches. Our findings initially confirmed our seventh hypothesis that the likelihood of finding theoretically valid moderating effects is progressively related to the degree of match between demands, resources, and outcomes. Indeed, the percentage of valid triple-match interactions was higher than the percentages for valid double-match and non-match interactions – in total as well as in both subgroups.

Furthermore, there was a significant positive association between the regression weights and the degree of match, which indicates that a higher degree of match is associated with stronger regression weights of the interactions. The explanatory theoretical mechanism here is that a match or congruence between two or more phenomena promotes vitality and health because it facilitates and increases the likelihood of functional coping when dealing with demands [55]. Therefore, the present study, showing that the TMP holds in two relatively large subgroups of employees and students in a university context, adds to the relevance and predictive validity of the DISC Model, and brings it closer to generalization.

### 4.2. Strengths and Limitations

This study has some strengths and limitations. An obvious strength was that we were able to examine and test the DISC Model’s relevance, validity, and generalizability in a university context, composing of two different types of groups (i.e., both employees and students). To our knowledge, this has not been done before. A first limitation concerns the cross-sectional study design as well as its reliance on self-report measures only. Though we suggested a particular causal order of the study variables, other causal directions or even reciprocal relations are also possible. For instance, fatigue might lead to cognitive and behavioral withdrawal reactions which in turn, lead to a lack of workplace social support [56]. Similarly, individuals with reduced vitality may recall more demands and fewer resources so that they report more demands and less support than was actually available to them [57]. Longitudinal survey studies should investigate these kinds of associations more profoundly. On the other hand, cross-sectional research designs are still necessary and pivotal in replication research [58]. A second limitation is that common method variance (CMV) due to using self-report data may have played a role, although research studies have shown that this influence is not as high as commonly believed [59,60]. We tried to minimize CMV by assessing the outcomes with different response formats and anchors compared to the predictor variables [61]. Nevertheless, multi-source and/or multi-method studies are recommended to deal with this kind of bias. Third, we did not control for students’ paid employment as a potential confounding factor for student stress. This should ideally be taken into account in future studies about students’ work stress [16]. A final remark it that the TMP is a probabilistic principle [28]. This raises questions of how to determine the distribution of this probabilistic event and whether our results are in line with this theoretical distribution. However, many studies are needed to statistically address this issue, which will only become possible when enough data are available for a meta-analysis. Currently, we can only say that the existing evidence clearly favors some match in contrast to no match at all.

### 4.3. Future Research

A first avenue for future research could be the long-term relation between demands and resources in the prediction of vitality and fatigue. Longitudinal DISC studies covering large and multiple time intervals are needed to investigate homeostatic regulation processes of match and non-match over time. Second, though already more specific than general concepts of “demands” and “resources”, the measures used in this study still are quite general in nature. Future research using more sector-specific or occupation-specific measures to assess demands and resources (e.g., focusing on staff’s research-related demands and resources, bureaucratic demands and resources, or exam demands and resources) can shed more light on their particular interplay [50,62]. A third avenue for future research could be the potential differential effects of different types of health and performance outcomes. The question remains if (and why) certain types of outcomes are more sensitive to matching demand/resource interactions than others? Finally, our research has mainly focused on processes occurring at work or study. Experiences and events happening *off* the job or study may also be related to vitality and fatigue. For instance, research on recovery has shown that recovery experiences during non-work time interact with demands and resources, too (e.g., [63,64]). Gaining knowledge about how both demands and resources as well as recovery are related to these outcomes is highly relevant for university staff and students as well.

### 4.4. Practical Implications

The current study shows the important role of matching resources in the relation between on the one hand demands and vitality and fatigue on the other in university staff and students. More specifically, the idea of match can be considered to be a leitmotiv for job or study redesign. Professionals in charge of job redesign will often have encountered difficulties in reducing or altering demands to combat fatigue. Enhancing particular resources instead enables people to deal with high demands at work or study, especially if these demands are matched with the right type of resources. Therefore, university management should consider the option to provide adequate matching job and study resources to individuals to prevent the risks associated with very high job and study demands. 

More specifically, employees who often encounter high cognitive demands (e.g., very complex problems, too many complex issues to handle, time-consuming bureaucratic processes) and who simultaneously have low cognitive resources (e.g., lack of job control, lack of job variety, not having a say in bureaucratic processes and choices, lack of administrative support, or little access to adequate information) are at risk of reporting high symptoms of cognitive and emotional fatigue. Enhancing the availability, visibility and actual use of cognitive resources will enable them to meet their high cognitive demands more adequately [45,65]. In this respect, job crafting can be an effective means for employees to shape their own job demands and resources to a large extent (e.g., [66]). Job crafting can be considered to be proactive employee behavior consisting of resource-seeking, challenge-seeking, and demand-reducing that employees engage in to create a better fit with their personal abilities and needs.

Similarly, students who are regularly confronted with high cognitive study demands (e.g., difficult assignments or exams, too many courses simultaneously, high grades/performance pressure) as well as low cognitive study resources (e.g., bad rosters, non-explanatory tutorials, low quality of handouts or books, lack of study control, tight deadlines) are in danger of reporting high symptoms of cognitive fatigue. Improving the availability of cognitive study resources, such as the implementation of more tutorial classes, better rostering, more control, appropriate deadlines or better (access to) exam material, will assist them to tackle efficiently their cognitive study demands and, consequently, reduce their cognitive fatigue symptoms. Moreover, a well-balanced match between cognitive demands and resources might increase their cognitive vitality, too. Furthermore, students who are opposed to high emotional demands (e.g., conflicts or impolite colleagues, lecturers or parents) and have low emotional resources such as emotional support, seem to be at risk of reporting more symptoms of emotional fatigue. Increasing emotional study resources such as emotional support from other students, lecturers, student advisors, or parents might lead to less symptoms of emotional fatigue.

## 5. Conclusions

In conclusion, this study gives insight into the specific (matching) combinations between demands and resources in the prediction of vitality and fatigue in a university context. Furthermore, it adds to the empirical evidence in favor of the DISC Model and its triple-match principle. Our findings indicate that the importance of matching resources should not be underestimated, although this conclusion seems to be more valid for fatigue than for vitality as an outcome. In general, the question of whether a particular demand and resource match is not easy to answer. It usually depends on the context. Cognitive and emotional demand/resource interactions seem to be important in an academic environment, whereas physical demands and physical resources are less salient in such a context. However, this applies to every work stress theory, because every theory represents an abstraction of reality. The DISC Model represents such an abstraction, too, but our findings prove this abstraction to be very promising.

In a sustainable work system, key building blocks are the preservation of nonrenewable resources and the regeneration of renewable resources [67]. Our study adds to this in a way that a sustainable work system is also about maintaining a healthy balance between sufficient, matching resources on the one hand, and the job or study demands on the other. In the end, universities should take care of human sustainability, so that they attract and retain vital and healthy staff and students.

## Figures and Tables

**Table 1:** Descriptive statistics and Pearson zero-order correlations among study variables (*n* = 894).

Variables	M	SD	1	2	3	4	5	6	7	8	9	10	11	12	13	14	15
1. Age	27.47	12.49															
2. Gender	0.42	0.49	0.11 **														
3. Education	4.76	1.44	0.40 **	0.01													
4. Cognitive Demands	3.50	0.62	0.02	0.01	0.12 **												
5. Emotional Demands	2.22	0.73	0.07 *	0.09 *	0.06	0.42 **											
6. Physical Demands	1.50	0.68	−0.07 *	−0.06	−0.07 *	0.21 **	0.42 **										
7. Cognitive Resources	3.68	0.69	0.08 *	0.01	0.09 **	0.08 *	0.19 **	−0.05									
8. Emotional Resources	3.29	0.81	0.08 *	0.14 **	0.03	0.12 **	−0.12 **	−0.01	0.47 **								
9. Physical Resources	3.32	1.21	0.04	0.04	0.01	0.08 *	−0.09 **	0.00	0.34 **	0.34 **							
10. Cognitive Fatigue	2.01	0.84	−0.15 **	0.03	−0.04	0.09 *	0.28 **	0.12 **	−0.11 **	−0.13 **	0.02						
11. Emotional Fatigue	1.91	0.83	−0.17 **	0.08 *	−0.01	0.19 **	0.38 **	0.13 **	−0.15 **	−0.20 **	0.02	0.68 **					
12. Physical Fatigue	1.56	0.68	−0.02	0.09 *	0.05	0.12 *	0.27 **	0.19 **	−0.07 *	−0.10 **	0.01	0.51 **	0.57 **				
13. Cognitive Liveliness	5.04	1.13	0.15 **	−0.02	0.04	0.15 **	−0.06	0.03	0.28 **	0.32 **	0.09 **	−0.36 **	−0.38 **	−0.21 **			
14. Emotional Energy	5.30	1.11	0.12 **	0.21 **	0.06	0.14 **	−0.02	−0.03	0.22 **	0.38 **	0.14 **	−0.15 **	−0.17 **	−0.07 *	0.49 **		
15. Physical Strength	4.69	1.21	0.28 **	−0.03	−0.07 *	0.08 *	−0.21 **	0.01	0.30 **	0.32 **	0.07 *	−0.42 **	−0.52 **	−0.35 **	0.57 **	0.39 **	

* significant at *p* < 0.05; ** significant at *p* < 0.01 (two-tailed).

**Table 2:** Hierarchical regression models of vitality and fatigue with triple-match and double-match (“common kind”) interactions (Employees; *n* = 397).

Source	Dependent Variable											
	Vitality						Fatigue					
	Cognitive Liveliness		Emotional Energy		Physical Strength		Cognitive		Emotional		Physical	
	B	SE	B	SE	B	SE	B	SE	B	SE	B	SE
Control Variables												
Age	0.02 **	0.00	0.01 **	0.00	0.01 **	0.00	−0.01 **	0.00	−0.01 **	0.00	−0.00	0.00
Gender	0.10	0.11	0.28 **	0.10	−0.02	0.11	0.04	0.08	0.05	0.07	0.05	0.07
Education	0.09 *	0.05	0.06	0.05	−0.07	0.05	0.06	0.03	0.07 *	0.03	−0.00	0.03
Demands and Resources												
Cognitive demands	0.19 **	0.06	0.08	0.06	0.20 **	0.06	−0.11 *	0.04	−0.03	0.04	−0.02	0.04
Emotional demands	−0.14 *	0.06	−0.02	0.06	−0.31 **	0.07	0.22 **	0.04	0.25 **	0.04	0.11 **	0.04
Physical demands	0.07	0.06	−0.08	0.06	0.13 *	0.06	0.08	0.04	0.04	0.04	0.14 **	0.04
Cognitive resources	0.21 **	0.06	0.11	0.06	0.21 **	0.07	0.00	0.05	−0.05	0.04	0.02	0.04
Emotional resources	0.31 **	0.06	0.29 **	0.06	0.33 **	0.07	−0.12 *	0.05	−0.16 **	0.04	−0.13 **	0.04
Physical resources	−0.15 **	0.05	−0.05	0.06	−0.15 **	0.06	0.11 *	0.04	0.13 **	0.04	0.08 *	0.04
Interaction Effects												
Cogn. demands × cogn. resources			−0.03 D	0.03			−0.06 * T	0.02	−0.06 ** D	0.02	−0.05 * D	0.02
Emo. demands × emo. resources			0.03 T	0.05			−0.05 D	0.04	−0.05 T	0.04	−0.02 D	0.03
Phys. demands × phys. resources			−0.15 * D	0.07			0.12 * D	0.05	0.11 * D	0.05	0.09 T	0.05
Model test	R^2^ = 0.28		R^2^ = 0.25		R^2^ = 0.24		R^2^ = 0.20		R^2^ = 0.30		R^2^ = 0.16	
	F (9, 387) = 16.61 **		F (12, 384) = 10.86 **		F (9, 387) = 13.91 **		F (12, 384) = 8.17 **		F (12, 384) = 13.78 **		F (12, 384) = 5.90 **	
Delta R^2^ interaction model			0.02				0.04		0.04		0.02	

Note: Cogn. = cognitive; Emo. = emotional; Phys. = physical; T = triple-match; D = double-match. * *p* < 0.05; ** *p* < 0.01 (two-tailed).

**Table 3:** Hierarchical regression models of vitality and fatigue with triple-match or double-match (“common kind”) interactions (Students; *n* = 497).

Source	Dependent Variable											
	Vitality						Fatigue					
	Cognitive Liveliness		Emotional Energy		Physical Strength		Cognitive		Emotional		Physical	
	B	SE	B	SE	B	SE	B	SE	B	SE	B	SE
Control Variables												
Age	0.01	0.01	0.00	0.01	−0.00	0.01	−0.00	0.01	−0.00	0.01	0.00	0.01
Gender	−0.11	0.09	0.43 **	0.09	−0.16	0.10	0.05	0.07	0.24 **	0.07	0.19 **	0.06
Education	−0.06	0.05	0.01	0.05	−0.09	0.05	0.01	0.04	0.03	0.03	0.06 *	0.03
Demands and Resources												
Cognitive demands	0.16 **	0.05	0.12 *	0.05	0.16 **	0.06	−0.04	0.04	0.04	0.04	−0.01	0.03
Emotional demands	−0.10	0.06	−0.12 *	0.06	−0.36 **	0.06	0.23 **	0.05	0.27 **	0.04	0.13 **	0.04
Physical demands	0.07	0.05	0.03	0.05	0.11 *	0.05	−0.09 *	0.04	−0.09 *	0.04	0.02	0.03
Cognitive resources	0.16 **	0.06	−0.03	0.06	0.22 **	0.06	−0.17 **	0.05	−0.13 **	0.04	−0.08 *	0.04
Emotional resources	0.24 **	0.05	0.36 **	0.05	0.20 **	0.06	−0.10 *	0.04	−0.18 **	0.04	−0.07 *	0.03
Physical resources	0.12 *	0.06	0.00	0.05	−0.03	0.06	0.03	0.05	0.07	0.04	−0.01	0.04
Interaction Effects												
Cogn. demands × cogn. resources	0.08 * T	0.03					−0.06 * T	0.03	−0.03 D	0.02	−0.03 D	0.02
Emo. demands × emo. resources	0.06 D	0.05					−0.04 D	0.04	−0.11 ** T	0.04	−0.04 D	0.03
Phys. demands × phys. resources	0.18 * D	0.07					−0.08 D	0.05	0.01 D	0.05	−0.05 T	0.04
Model test	R^2^ = 0.15		R^2^ = 0.18		R^2^ = 0.17		R^2^ = 0.15		R^2^ = 0.25		R^2^ = 0.13	
	F (12, 484) = 7.31 **		F (9, 487) = 10.84 **		F (9, 487) = 11.37 **		F (12, 484) = 6.84 **		F (12, 484) = 13.20 **		F (12, 484) = 5.86 **	
Delta R^2^ interaction model	0.05						0.03		0.03		0.02	

Note: Cogn. = cognitive; Emo. = emotional; Phys. = physical; T = triple-match; D = double-match. * *p* < 0.05; ** *p* < 0.01 (two-tailed).

**Table 4:** Summary of analyses of interaction effects with different patterns of match in both subgroups as well as in the total sample.

Interaction Pattern	Valid Interactions	Reversed Interactions	Tested Interactions	Ratio of Valid Interactions/Interactions Tested (%)
Employees (*n* = 397) Triple-match Double-match Non-match	1 3 0	0 5 1	6 36 12	16.7% 8.3% 0.0%
Students (*n* = 497) Triple-match Double-match Non-match	3 5 1	0 0 1	6 36 12	50.0% 13.9% 8.3%
Total (*n* = 894) Triple-match Double-match Non-match	4 8 1	0 5 2	12 72 24	33.3% 11.1% 4.2%

## Appendix A

**Table A1:** Hierarchical regression models of vitality and fatigue with non-match or double-match (“extended kind”) interactions (Employees; *n* = 397).

Source	Dependent Variable											
	Vitality						Fatigue					
	Cognitive Liveliness		Emotional Energy		Physical Strength		Cognitive		Emotional		Physical	
	B	SE	B	SE	B	SE	B	SE	B	SE	B	SE
Control Variables												
Age	0.02 **	0.00	0.01 **	0.00	0.01 *	0.00	−0.01 **	0.00	−0.01 **	0.00	−0.00	0.00
Gender	−0.09	0.11	0.31 **	0.10	−0.07	0.12	0.05	0.08	0.06	0.07	0.05	0.07
Education	0.09 *	0.05	0.06	0.05	−0.10	0.05	0.07 *	0.03	0.08 **	0.03	0.01	0.03
Demands and Resources												
Cognitive demands	0.19 **	0.06	0.12 *	0.06	0.17 *	0.07	−0.11 *	0.05	−0.04	0.04	−0.03	0.04
Emotional demands	−0.14 *	0.06	−0.01	0.06	−0.32 **	0.07	0.23 **	0.05	0.26 **	0.04	0.11 **	0.04
Physical demands	0.07	0.06	−0.06	0.06	0.09	0.07	0.07	0.04	0.04	0.04	0.15 **	0.04
Cognitive resources	0.21 **	0.06	0.14 *	0.06	0.16 *	0.07	0.01	0.05	−0.05	0.04	0.01	0.04
Emotional resources	0.31 **	0.06	0.30 **	0.06	0.30 **	0.07	−0.09	0.05	−0.13 **	0.04	−0.10 *	0.04
Physical resources	−0.15 **	0.05	0.01	0.05	−0.18 **	0.06	0.07	0.04	0.10 **	0.04	0.05	0.03
Interaction effects												
Cogn. demands × emo. resources					−0.07 N	0.06	0.01 D	0.04	−0.02 D	0.04	−0.07 N	0.03
Cogn. demands × phys. resources					0.12 D	0.07	−0.06 D	0.05	−0.06 N	0.04	−0.05 D	0.04
Emo. demands × cogn. resources					0.00 N	0.06	−0.09 * D	0.04	−0.05 D	0.04	−0.01 N	0.04
Emo. demands × phys. resources					−0.16 ** D	0.06	0.08 * N	0.04	0.09 * D	0.04	0.06 D	0.04
Phys. demands × cogn. resources					−0.07 D	0.07	0.01 D	0.05	0.03 N	0.04	0.07 D	0.04
Phys. demands × emo. resources					0.01 D	0.07	−0.00 N	0.05	−0.01 D	0.05	0.02 D	0.04
Model test	R^2^ = 0.28		R^2^ = 0.23		R^2^ = 0.27		R^2^ = 0.20		R^2^ = 0.29		R^2^ = 0.16	
	F (9, 387) = 16.61 **		F (9, 387) = 13.38 **		F (15, 381) = 9.52 **		F (15, 381) = 6.31 **		F (15, 381) = 10.40 **		F (15, 381) = 4.91 **	
Delta R^2^ interaction model					0.03		0.04		0.03		0.03	

Note: Cogn. = cognitive; Emo. = emotional; Phys. = physical; N = non-match; D = double-match. * *p* < 0.05; ** *p* < 0.01 (two-tailed).

**Table A2:** Hierarchical regression models of vitality and fatigue with non-match or double-match (“extended kind”) interactions (Students; *n* = 497).

Source	Dependent Variable											
	Vitality						Fatigue					
	Cognitive Liveliness		Emotional Energy		Physical Strength		Cognitive		Emotional		Physical	
	B	SE	B	SE	B	SE	B	SE	B	SE	B	SE
Control Variables												
Age	0.01	0.01	0.00	0.01	−0.00	0.01	−0.00	0.01	−0.01	0.00	0.00	0.01
Gender	−0.12	0.09	0.42 **	0.09	−0.16	0.10	0.05	0.07	0.23 **	0.07	0.19 **	0.06
Education	−0.06	0.05	−0.00	0.05	−0.09	0.05	0.00	0.04	0.02	0.03	0.06 *	0.03
Demands and Resources												
Cognitive demands	0.16 **	0.05	0.14 **	0.05	0.16 **	0.06	−0.04	0.04	0.06	0.04	−0.01	0.03
Emotional demands	−0.10	0.06	−0.10	0.06	−0.36 **	0.05	0.22 **	0.05	0.31 **	0.04	0.14 **	0.04
Physical demands	0.07	0.05	0.01	0.05	0.11 *	0.06	−0.09 *	0.04	−0.10 **	0.04	0.01	0.03
Cognitive resources	0.17 **	0.06	−0.01	0.06	0.22 **	0.06	−0.18 **	0.05	−0.10 *	0.04	−0.08 *	0.04
Emotional resources	0.24 **	0.05	0.37 **	0.05	0.20 **	0.06	−0.10 *	0.04	−0.16 **	0.04	−0.06	0.03
Physical resources	0.09	0.06	0.04	0.05	−0.03	0.06	0.05	0.04	0.08	0.04	0.00	0.03
Interaction effects												
Cogn. demands × emo. resources	−0.02 D	0.05	−0.04 D	0.05			−0.02 D	0.04			−0.03 N	0.03
Cogn. demands × phys. resources	0.00 D	0.06	0.04 N	0.06			−0.01 D	0.05			−0.02 D	0.04
Emo. demands × cogn. resources	0.13 * D	0.06	0.14 * D	0.06			−0.10 * D	0.05			0.02 N	0.04
Emo. demands × phys. resources	0.13 * N	0.06	0.15 * D	0.06			−0.03 N	0.05			−0.00 D	0.04
Phys. demands × cogn. resources	0.00 D	0.06	−0.12 * N	0.06			−0.02 D	0.05			−0.03 D	0.04
Phys. demands × emo. resources	0.08 N	0.05	−0.01 D	0.05			−0.02 N	0.04			−0.06 D	0.03
Model test	R^2^ = 0.16		R^2^ = 0.20		R^2^ = 0.17		R^2^ = 0.15		R^2^ = 0.22		R^2^ = 0.13	
	F (15, 481) = 6.05 **		F (15, 481) = 8.01 **		F (9, 487) = 11.37 **		F (15, 481) = 5.71 **		F (9, 487) = 15.43 **		F (15, 481) = 4.90 **	
Delta R^2^ interaction model	0.05		0.03				0.03				0.02	

Note: Cogn. = cognitive; Emo. = emotional; Phys. = physical; N = non-match; D = double-match. * *p* < 0.05; ** *p* < 0.01 (two-tailed).
